# Patterns of Childhood Obesity Prevention Legislation in the United States

**Published:** 2007-06-15

**Authors:** Ross C Brownson, Tegan K Boehmer, Debra Haire-Joshu, Mariah L Dreisinger

**Affiliations:** Saint Louis University School of Public Health; Saint Louis University School of Public Health, St. Louis, Mo. Tegan K. Boehmer is now affiliated with the Epidemic Intelligence Service at the Centers for Disease Control and Prevention in Atlanta, Ga; Saint Louis University School of Public Health, St. Louis, Mo; Saint Louis University School of Public Health, St. Louis, Mo

## Abstract

**Introduction:**

Because of the public's growing awareness of the childhood obesity epidemic, health policies that address obesogenic environments by encouraging healthy eating and increased physical activity are gaining more attention. However, there has been little systematic examination of state policy efforts. This study identified and described state-level childhood obesity prevention legislation introduced and adopted from 2003 through 2005 and attempted to identify regional geographic patterns of introduced legislation.

**Methods:**

A scan of legislation from all 50 states identified 717 bills and 134 resolutions that met study inclusion criteria. Analyses examined patterns in the introduction and adoption of legislation by time, topic area, and geography.

**Results:**

Overall, 17% of bills and 53% of resolutions were adopted. The amount of legislation introduced and adopted increased from 2003 through 2005. The topic areas with the most introduced legislation were school nutrition standards and vending machines (n = 238); physical education and physical activity (n = 191); and studies, councils, or task forces (n = 110). Community-related topic areas of walking and biking paths (37%), farmers' markets (36%), and statewide initiatives (30%) had the highest proportion of bills adopted, followed by model school policies (29%) and safe routes to school (28%). Some regional geographic patterns in the introduction of legislation were observed. There was no statistical association between state-level adult obesity prevalence and introduction of legislation.

**Conclusion:**

Public health and health policy practitioners can use this information to improve advocacy efforts and strengthen the political climate for establishing childhood obesity prevention legislation within state governments. Expanded surveillance (including standardized identification and cataloging) of introduced and adopted legislation will enhance the ability to assess progress and identify effective approaches. Future policy research should examine determinants, implementation, and effectiveness of legislation to prevent childhood obesity.

## Introduction

In the United States, the prevalence of overweight and obesity has been on a steady rise in all sex, age, race, and education subgroups for the past several decades (1-3). Between 1980 and 2000, the prevalence of childhood overweight (body mass index [BMI] ≥95th percentile) more than doubled among 2- through 11-year olds and tripled among 12- through 19-year olds (4). The problem of obesity among youths is particularly concerning because of the immediate and long-term risks to physical and psychosocial health (5). The rapid rise in obesity prevalence among both youths and adults is most likely attributable to factors in the physical, social, economic, and policy environments that influence diet and activity (6,7).

The Institute of Medicine (IOM) states that the goal of obesity prevention among youths is to create through directed social change an environment–behavior synergy that promotes energy balance (8). Thus, policies that address obesogenic environments by encouraging healthy eating and increased physical activity are gaining attention (9-12). Health policies, in the form of laws, regulations, organizational practices, and funding priorities, have a substantial impact on the health and well-being of the population and have been used in past and recent history to address important public health issues (e.g., tobacco control, nutritional deficiencies, highway safety) (8,10). Examples of regulatory and legislative actions that focus on a population approach of obesity reduction include requiring labeling of nutritional content of food served in restaurants, imposing advertising restrictions, mandating school nutrition and physical education programs, regulating competitive foods and vending machine contracts in schools, enforcing mixed-use zoning, and improving opportunities or incentives for nonmotorized transportation (11,13).

In the United States, much of the authority for public health policy lies at the state level — through the legislative and regulatory actions taken by the state government and the manner in which the state constitution imparts authority to local governments (14). Successful health policy depends on three criteria: 1) existence of a sufficient evidence base, 2) development of effective coalitions, and 3) commitment of policy makers (10). Much of the political activity surrounding obesity policy has occurred within state legislatures rather than the federal government. Within the past few years, many states have introduced legislation (formal written codes such as bills and resolutions) that focuses on obesity prevention in youth, typically through increasing physical activity and improving nutrition within the school and community environments.

There has been little systematic examination of current state-level policy efforts in obesity prevention. A recently developed framework for policy research related to physical activity describes four types of studies: 1) identification of relevant policies, 2) recognition of determinants of establishing policy, 3) development and implementation of policy, and 4) examination of policy outcomes (15). This framework also specifies the setting of policy research in terms of scale (e.g., state-level policy) and sector (e.g., school, community) (15). This study addresses the first phase of the framework. The aim of this study was to identify and describe introduced and adopted state-level legislation relevant to the prevention of childhood obesity in all 50 states from 2003 through 2005.

## Methods

### Terminology

This study of childhood obesity prevention legislation includes both bills and resolutions. A *bill* is a proposed new law or amendment to an existing law that is presented to the legislature for consideration. To become law, bills require approval by both chambers of the legislature and by the governor. (Bills can be enacted with or without the governor's signature as long as they are not vetoed.) Bills may appropriate money, prescribe fees or penalties, repeal existing law, or take other action. A *resolution* is a formal expression of the will, opinion, or direction of one or both chambers of the legislature on a matter of public interest. Simple resolutions require approval only by one chamber; concurrent and joint resolutions require approval by both chambers. In general, resolutions require no action by the governor and do not have the force of law.

Different terminology is used to describe the final approval of a bill or resolution in the legislative process. For example, *enact* means to establish by law and refers to the final approval of bills, whereas a*dopt* means to approve or endorse and is usually applied to amendments and resolutions (but not bills). To simplify the terminology used in this study, *adoption* was defined as a favorable final action (i.e., approval in the last stage of the legislative process) for both resolutions and bills. Consistent with the definitions provided above, adoption was defined differently for simple resolutions (approved in the chamber of origin), joint and concurrent resolutions (approved in both chambers), and bills (approved in both chambers and enacted into law).

### Identification of relevant legislation

We used a legislative database created by Netscan's Health Policy Tracking Service (HPTS) (16) to identify state legislation affecting nutrition, physical activity, and obesity prevention introduced in all 50 states from 2003 through 2005. HPTS performed a legislative scan for 2003 and 2004 using the same search criteria that were previously developed for their 2005 report on state nutrition, activity, and obesity legislation (17). HPTS performed separate searches on 24 topic areas (e.g., farmers' markets, nutrition standards and vending machines, BMI reporting, safe routes to school), so it was possible for a single bill or resolution to be listed in more than one topic area.

The legislative scan identified 1149 bills and resolutions (including simple, joint, and concurrent resolutions) introduced from January 1, 2003, through December 31, 2005. We excluded bills that were merged with or substituted by a similar bill that was subsequently enacted (n = 35), resulting in 965 bills and 149 resolutions for further consideration. We reduced the number of topic areas from 24 to 18 by combining similar categories and categories with small numbers. The 18 topic areas were categorized as relevant or irrelevant to childhood obesity prevention. Four topic areas were excluded because of irrelevance: 1) labeling of genetically modified food products, 2) insurance coverage of gastric bypass surgery, 3) Medicaid coverage of obesity-related treatments, and 4) restrictions on civil liability lawsuits related to obesity and food consumption.

The 14 relevant topic areas were further categorized as school-related or community-related. The 813 bills and 144 resolutions within these topic areas were examined in more detail to ensure their applicability to childhood obesity (yes or no) and direction of health impact (positive, negative, or unsure). The task of coding was divided among four members of the research team. Eighty bills were coded in duplicate to assess interrater agreement. Agreement between raters was 89% for applicability (n = 80) and 94% for health impact (n = 63) (health impact was coded only for applicable bills). We excluded from further consideration bills and resolutions that were coded as not applicable (e.g., specific to senior citizens, concerning sex education in schools) or as having a negative health impact (e.g., repealing of BMI reporting, allowing exemptions for physical education). After removal of 78 bills and 10 resolutions that were not applicable and 18 bills with a negative health impact, this study reviewed 717 bills and 134 resolutions. Legislative history was reviewed to determine whether each bill or resolution was adopted as of December 31, 2005.

### Determination of legislative patterns

A descriptive analysis was performed to examine patterns in the introduction and adoption of legislation by time, topic area, and geography. Patterns over time were described by comparing data for 2003 and 2005. Because of differences in the frequency and length of legislative sessions, fewer bills and resolutions are introduced in even years. (Six states have biennial sessions that meet only in odd years; among states that meet annually, 25 have 2-year sessions that begin in odd years [e.g., 2003–2004].)

The number introduced and adopted and percentage adopted were calculated separately for bills and resolutions for each of the 14 relevant topic areas and for each of the 50 states. In addition to quantity, the number of topic areas covered (possible range, 0-14) through introduced and adopted legislation was assessed to measure the breadth of approaches addressed within each state.

Next, we examined the geographical patterns of introduced legislation and topic areas covered by introduced legislation and compared them with obesity prevalence. Three U.S. maps were created with each variable of interest categorized into quartiles. Childhood obesity prevalence estimates were not available for all 50 states, so we used adult obesity prevalence from the 2004 Behavioral Risk Factor Surveillance System (18) as an indicator for childhood obesity prevalence. The association between adult obesity prevalence and childhood obesity legislation (number of introduced bills and resolutions) was examined dichotomously (low and high) using Pearson chi-square testing and was examined by quartiles (1 to 4) and rank order (1 to 50) using Spearman rank correlation. Analyses were performed using SPSS version 14.0 (SPSS Inc, Chicago, Ill).

## Results

The 14 childhood obesity prevention topic areas are described in Table 1. During the 3-year study period, 123 (17%) of the 717 introduced bills were adopted, and 71 (53%) of the 134 introduced resolutions were adopted. From 2003 through 2005, there was an increase in the annual number of bills introduced (199 to 339) and adopted (40 to 55); however, the proportion adopted decreased from 20% to 16% (data not shown). Similarly, the annual number of resolutions introduced from 2003 through 2005 increased (40 to 55) and the number adopted remained steady (25 to 23), resulting in a decrease in the proportion adopted from 62% to 42% (data not shown).

The likelihood of introduction and adoption varied by topic area (Table 2). The topic areas with the greatest number of introduced bills and resolutions were school nutrition standards and vending machines (n = 238); physical education and physical activity (n = 191); and studies, councils, or task forces (n = 110). Community-related topic areas of walking and biking paths (37%), farmers' markets (36%), and statewide initiatives (30%) had the greatest proportion of bills adopted, followed by model school policies (29%) and safe routes to school (28%). School nutrition standards and vending machines had the lowest proportion of bills adopted (13%), possibly because of the large number of bills and resolutions introduced (i.e., average of nearly five bills or resolutions introduced per state during the 3-year period). None of the bills related to snack and soda taxes or restaurant menu and product labeling were adopted.

The number of bills and resolutions introduced and adopted and the number of topic areas covered are provided by state (Table 3). The number of bills introduced ranged from 0 (Wyoming) to 51 (New York) with a median of 11. The number of bills adopted ranged from 0 (12 states) to 10 (California and Illinois) with a median of 2, and the proportion adopted ranged from 0% to 75% (Colorado). The number of resolutions introduced ranged from 0 (18 states) to 23 (Hawaii) with a median of 1. The number of resolutions adopted ranged from 0 (22 states) to 12 (California) with a median of 1, and the proportion adopted ranged from 0% to 100%. The number of topic areas addressed through introduced legislation (bills and resolutions combined) was highest for Connecticut, Illinois, Massachusetts, New York, and Texas (median = 8). The states with the highest number of topic areas adopted were California, Illinois, Louisiana, and New York (median = 3).

No statistical association between adult obesity prevalence and introduced legislation was observed. However, some general geographic patterns were observed (Figure). Slightly more than half of states (n = 28) showed concordance between obesity prevalence and amount of introduced legislation (when both variables were dichotomized as either low or high). Of the 14 states with below-average obesity prevalence and low legislative activity, 7 were in the mountain region (Arizona, Colorado, Idaho, Montana, Nevada, Utah, and Wyoming). Ten of the 14 states with above-average obesity prevalence and high legislative activity were in the south central or south Atlantic regions (Alabama, Arkansas, Georgia, Maryland, Mississippi, North Carolina, Oklahoma, Tennessee, Texas, and West Virginia). In contrast, 7 of the 12 nonconcordant states with above-average obesity prevalence and low legislative activity were in the midwest region (Iowa, Indiana, Kansas, North Dakota, Nebraska, South Dakota, and Wisconsin), whereas the 10 states with below-average obesity prevalence and high legislative activity were spread throughout the Pacific west (California, Hawaii, and Washington), mountain (New Mexico), midwest (Illinois and Minnesota), and northeast (Connecticut, Massachusetts, New York, and Rhode Island) regions.

**Figure 1:**
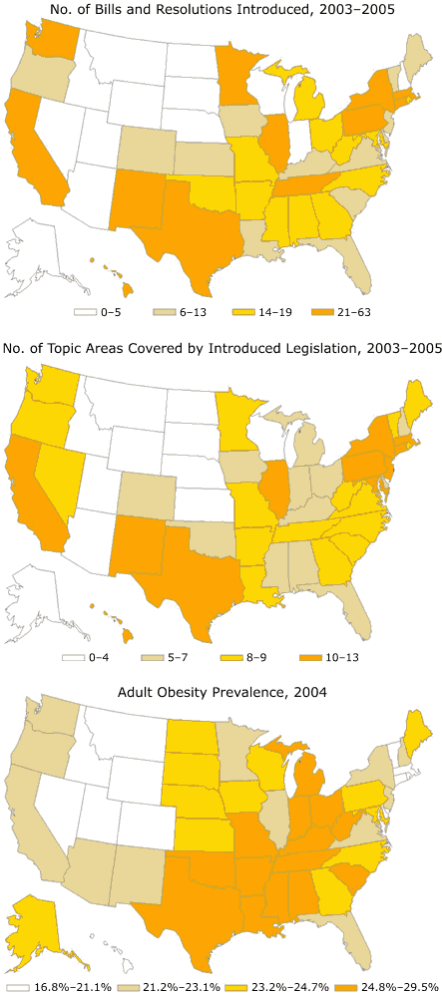
Number of bills and resolutions introduced and number of topic areas covered by introduced legislation, 2003–2005, and prevalence of adult obesity, 2004, United States.

**Table:** Bills and Resolution Introduced, 2003–2005

**Table:** Topic Areas Covered by Introduced Legislation, 2003–2005

**No. Topic Areas Covered**	**No. States**
0-4	11
5-7	12
8-9	16
10-13	11

**Table:** Adult Obesity Prevalence, 2004

**Percent Obese**	**No. States**
16.8-21.1	12
21.2-23.1	12
23.2-24.7	12
24.8-29.5	14

## Discussion

This study is among the first to systematically identify, describe, and assess patterns in legislation to prevent childhood obesity. The findings of this study provide useful information for public health and health policy practitioners and suggest directions for future policy research related to obesity prevention. Study findings and recommendations are summarized below according to phases of the policy research framework proposed by Schmid et al (15).

### Identification of relevant policies

We found that the number of bills and resolutions introduced and adopted increased from 2003 through 2005. Given this short time frame, there is a need for continued monitoring of nutrition, physical activity, and obesity prevention legislation to assess trends over time. Legislative tracking services and surveillance systems will be useful in all phases of policy research. As part of surveillance, it is important to develop a standardized method for identifying and cataloging legislation. This will likely prove to be a difficult task given the wide range of topic areas that fall under the umbrella of obesity prevention (e.g., urban development, transportation, farmers' markets, task forces, school nutrition, advertising).

A few tracking resources are available to the public for monitoring the introduction of obesity prevention legislation, including the Centers for Disease Control and Prevention's (CDC's) Nutrition and Physical Activity Legislative Database (19) and the National Conference of State Legislature's (NCSL's) Healthy Community Design Legislation Database (20). However, we found low concordance between the HPTS, CDC, and NCSL legislative databases in terms of the number of bills introduced and how they were categorized. A single, standardized database of all introduced legislation will assist with future research for identification of relevant policies and determinants of policy adoption.

### Determinants of establishing policy

We found that legislation within certain topic areas was more likely to be adopted than others. Additional research is needed to identify characteristics of bills that are adopted. For example, it may be that legislation related to statewide initiatives, studies, and task forces may be easier to pass because of the limited amount of resources necessary to implement such laws, and resource-intensive and revenue-restricting bills may be more difficult to pass. Understanding bill characteristics that are associated with adoption may assist with the development of model legislation and lead to more successful advocacy efforts.

Geographic comparisons also demonstrated wide variation among states in the amount of legislation introduced and the proportion of legislation adopted. Although regional geographic patterns were identified, no statistical link was found between legislative activity and adult obesity prevalence. Future research should examine why some states are more likely to introduce and adopt childhood obesity prevention legislation than other states. For example, state-level political, economic, and sociocultural factors may affect legislative priorities within state governments. As a follow-up to this study, we plan to conduct a quantitative, multilevel analysis to examine both bill-level and state-level factors associated with bill adoption. Another possibility is to conduct qualitative case studies of states that are considered high or low adopters.

### Development and implementation of policy

Certain topic areas (e.g., school nutrition, task forces) were more commonly introduced than others and may represent early steps in the development of obesity prevention policies. For example, vending machine restrictions were first considered and adopted in California, setting an example and providing momentum for other states to follow. Additionally, bills and resolutions related to statewide initiatives, studies, and task forces likely represent a first, capacity-building step in a process leading toward more comprehensive programs and policies. Future research should investigate the process of policy development as it relates to childhood obesity prevention. This may include establishing a way to measure a state's level of readiness for developing and implementing childhood obesity prevention legislation, as well as outlining stages of progress.

The extent to which evidence guides obesity prevention policies is also important to evaluate. Researchers often assume that evidence guides policy development. However, policymakers are influenced by multiple domains (e.g., social, media-related, economic). Assessing the extent to which current policy initiatives are guided in development by multiple forms of data and the role evidence plays in that process are critical in understanding effective evaluation of policy impact (21).

### Examination of policy outcomes

Surveillance of enacted legislation will promote research on policy quality, implementation, and effectiveness at achieving desired health outcomes. For example, the National Cancer Institute's State Cancer Legislative Database program maintains a public-use, searchable database of adopted legislation related to several types of cancer (e.g., breast, cervical, prostate, skin) as well as access to care, genetics, surveillance, and tobacco control (22). This database has been used in several analytic studies to evaluate the scope and quality of enacted policies and their impact on health behaviors and outcomes, such as youth access to tobacco and clean indoor air laws (23-26). As policies related to obesity prevention continue to be introduced and adopted, a database of enacted legislation should be developed to assist with future studies examining the impact of policy on outcomes related to energy balance.

### Limitations

The findings of this study are subject to at least four limitations. First, the limited time period of the study prohibited the examination of trends over time. Extension of the time period was not possible, and childhood obesity-related legislation introduced before 2003 was limited. Second, this study may slightly underestimate the proportion of bills adopted, because legislation introduced in 2005 may have been carried over and adopted in 2006 in the 25 states that have 2-year legislative sessions (2005–2006). Third, the quality of data in the HPTS legislative database depends upon information available from state Web sites. Therefore, some information about current bill status (adopted or not) may have been incomplete or out-of-date. Finally, the identification and categorization of bills within topic areas were based on HPTS search criteria, which likely differ from that of other agencies (e.g., CDC, NCSL). Unfortunately, none of the available legislative databases have been formally validated or compared with one other. As a result, the quality of the HPTS legislative database is unknown, both in terms of completeness (i.e., amount of legislation introduced) and accuracy (i.e., classification of legislation into topic areas).

### Implications for practice

This study is an initial attempt to develop policy-relevant data on childhood obesity. This information can be powerful in assessing progress, identifying effective approaches, and supporting advocacy efforts to address the problem. As such, there are several implications for public health practitioners:

- State and federal health officials should consider policy surveillance as an evaluation component of state plans to prevent obesity (27). Many states funded through CDC's Nutrition and Physical Activity Program have reported environmental changes through policy and legislation (28). To assess progress, states should consider monitoring policy development and implementation and, more importantly, effectiveness at achieving desired outcomes.
- Health policy and public health practitioners may be able to use this study as a starting point to identify more comprehensive policy approaches, as recommended by the IOM's childhood obesity report (8). A closer examination of states with a successful track record may lead to model policies and legislative approaches.
- Advocacy groups and interested legislators can use the information provided in this study to inform and motivate key stakeholders within the state government. For example, a simple description of a state's performance on obesity policy compared with other states (especially neighboring states) may improve political will and climate.

### Conclusion

The process of policy development involves three key criteria: 1) sufficient evidence base, 2) development of effective coalitions, and 3) commitment of policy makers (10). Although the knowledge base for successful programs and policies is limited (8) and movement toward social consensus and public action is just beginning (29), our study shows considerable adoption of legislation targeting childhood obesity. This suggests a growing desire and dedication among state legislators. Expanded policy surveillance (including standardized identification and cataloging) of introduced and adopted legislation will enhance our ability to track progress and identify effective approaches. Future policy research should examine the determinants, implementation, and effectiveness of legislation to prevent childhood obesity.

## Figures and Tables

**Table 1:** Description of Health Policy Tracking Service (HPTS) Legislative Topic Areas on Childhood Obesity Prevention

Topic Area	Description
School-related	
Nutrition standards and vending machines	Provide students with nutritional food and beverage items. Restrict access to vending machines and competitive foods. Regulate marketing of foods and beverages with minimal nutritional value. Report nutritional information and vending machine revenue.
Physical education and physical activity	Ensure schools have a physical education (PE) program. Set time and frequency requirements for PE classes. Restrict substitutions and waivers for PE. Promote physical activity in other classes.
Health education	Ensure schools include nutrition, physical activity, and obesity prevention in health education curriculum.
Curriculum for health and physical education classes	Govern changes to the state's curriculum relating to health, nutrition, and physical education. Require set hours of PE per week. Establish graduation requirements.
Local authority	Provide local districts the ability to set policies and create committees focused on reducing the prevalence of obesity among school children through regulation of nutrition and physical activity requirements.
Safe routes to school	Provide bicycle facilities (such as paths), sidewalks, crossing guards, and traffic-calming measures to enable children to bicycle or walk safely to school.
Body mass index reporting	Require or allow schools to measure, monitor, and report student's body mass index in conjunction with intervention strategies to help reduce childhood obesity.
Model school policies	Require state agencies or state education officials to develop model school policies relating to nutrition and physical education.
Community-related	
Studies, councils, or task forces	Establish a commission, committee, council, task force, or study to address obesity within schools or communities.
Farmers' markets	Support and make appropriations for farmers' market initiatives. Promote the implementation of locally grown nutritious foods in school systems.
Statewide initiatives	Establish initiatives, often through the state's department of health, to reduce the prevalence of obesity among residents statewide.
Walking and biking paths	Support (through appropriation and regulations) physical activity through the creation or maintenance of bicycle trails, walking paths, and sidewalks. Promote bicycle and pedestrian safety.
Soda and snack tax	Increase or establish a tax on snack and soft drink items. May use revenue to promote nutrition and health in schools.
Restaurant menu and product labeling	Regulates the labeling of nutrition content on food items. Requires restaurants to post nutritional information on menus/boards.

**Table 2:** Introduced^a^ and Adopted^b^ Legislation on Childhood Obesity Prevention, by Topic Area, United States, 2003–2005

Topic Area	Bills (N = 717)		Resolutions (N = 134)	

	No. Introduced	No. Adopted (%)	No. Introduced	No. Adopted (%)
School-related				
Nutrition standards and vending machines	213	27 (13)	25	9 (36)
Physical education and physical activity	165	26 (16)	26	14 (54)
Health education	68	12 (18)	5	3 (60)
Curriculum for health and physical education classes	61	9 (15)	7	2 (29)
Local authority	58	12 (21)	4	1 (25)
Safe routes to school	43	12 (28)	4	3 (75)
Body mass index reporting	37	8 (22)	2	1 (50)
Model school policies	14	4 (29)	1	1 (100)
Community-related				
Studies, councils, or task forces	68	11 (16)	42	15 (36)
Farmers' markets	87	31 (36)	3	3 (100)
Statewide initiatives	37	11 (30)	35	28 (80)
Walking and biking paths	46	17 (37)	2	2 (100)
Soda and snack tax	49	0 (0)	0 (0)	0 (0)
Restaurant menu and product labeling	25	0 (0)	0 (0)	0 (0)
Total^c^	717	123 (17)	134	71 (53)

a Bills and resolutions must have been introduced from January 1, 2003, through December 31, 2005, to be included in the study.

b Adoption of a bill or resolution must have taken place on or before December 31, 2005.

c Numbers and percentages do not add up to totals because some bills and resolutions were listed in more than one topic area.

**Table 3:** Introduced^a^ and Adopted^b^ Legislation on Childhood Obesity Prevention, by State, United States, 2003–2005

State^c^	Bills (N = 717)		Resolutions (N = 134)		Topic Areas	

	No. Introduced	No. Adopted (%)	No. Introduced	No. Adopted (%)	No. Introduced	No. Adopted
Alabama	11	2 (18)	6	4 (67)	5	4
Alaska	5	1 (20)	0	—	4	1
Arizona	5	2 (40)	0	—	4	2
Arkansas	14	5 (36)	1	1 (100)	9	6
California	38	10 (26)	13	12 (92)	11	8
Colorado	4	3 (75)	2	2 (100)	6	6
Connecticut	29	2 (7)	0	—	13	4
Delaware	5	0 (0)	2	2 (100)	5	3
Florida	11	2 (18)	1	1 (100)	6	2
Georgia	9	5 (56)	10	4 (40)	8	5
Hawaii	33	1 (3)	23	3 (13)	11	3
Idaho	3	0 (0)	0	—	4	—
Illinois	49	10 (20)	14	6 (43)	12	8
Indiana	4	0 (0)	1	0 (0)	7	—
Iowa	9	2 (22)	2	1 (50)	7	3
Kansas	5	1 (20)	1	1 (100)	2	2
Kentucky	12	1 (8)	0	—	5	2
Louisiana	10	6 (60)	3	3 (100)	8	8
Maine	9	1 (11)	2	1 (50)	9	5
Maryland	17	2 (12)	2	0 (0)	10	5
Massachusetts	42	4 (10)	0	—	12	3
Michigan	16	2 (12)	0	—	7	2
Minnesota	21	0 (0)	0	—	8	—
Mississippi	19	2 (11)	0	—	7	5
Missouri	12	0 (0)	3	0 (0)	9	—
Montana	3	0 (0)	1	1 (100)	3	1
Nebraska	4	0 (0)	1	0 (0)	4	—
Nevada	2	1 (50)	3	3 (100)	8	7
New Hampshire	4	1 (25)	0	—	5	2
New Jersey	9	3 (33)	1	1 (100)	10	3
New Mexico	46	8 (17)	10	5 (50)	11	7
New York	51	7 (14)	0	—	13	8
North Carolina	18	2 (11)	0	—	9	1
North Dakota	2	0 (0)	1	1 (100)	2	1
Ohio	14	2 (14)	0	—	7	1
Oklahoma	16	5 (31)	0	—	6	4
Oregon	12	1 (8)	0	—	8	1
Pennsylvania	15	4 (27)	10	6 (60)	11	3
Rhode Island	18	2 (11)	1	1 (100)	9	3
South Carolina	6	2 (33)	0	—	9	6
South Dakota	1	0 (0)	1	1 (100)	2	1
Tennessee	23	4 (17)	3	2 (67)	9	7
Texas	24	5 (21)	2	2 (100)	12	7
Utah	2	0 (0)	1	1 (100)	3	1
Vermont	9	1 (11)	1	1 (100)	8	4
Virginia	9	2 (22)	4	2 (50)	9	4
Washington	23	6 (26)	0	—	9	5
West Virginia	12	3 (25)	7	2 (29)	8	6
Wisconsin	2	0 (0)	1	1 (100)	2	1
Wyoming	0	—	0	—	0	—

a Bills and resolutions must have been introduced from January 1, 2003, through December 31, 2005, to be included in the study.

b Adoption of a bill or resolution must have taken place on or before December 31, 2005.

c Washington, D.C., was not included in the analysis.
